# Transradial vs transfemoral secondary access outcomes in transcatheter aortic valve replacement: an updated systematic review and meta-analysis

**DOI:** 10.1186/s43044-025-00706-3

**Published:** 2025-12-31

**Authors:** Hashim Ishfaq, Reyan Hussain Shaikh, Emaan Fatima, Mian Muinuddin Jamshed, Hamza Ishfaq, Abdulkareem Lukan, Hina Inam, Muhammad Bilal Ibrahim, Hafeez Shaka

**Affiliations:** 1 Medical College, Aga Khan University, Karachi, Pakistan; 2 Northwest Health-Porter, Valparaiso, USA; 3 Advocate Illinois Masonic Medical Center, Chicago, USA; 4 Department of Cardiothoracic Surgery, Aga Khan University Hospital, Karachi, Pakistan; 5 John H. Stroger, Jr. Hospital of Cook County, Chicago, USA

**Keywords:** Transcatheter aortic valve implantation, Femoral access, Radial access, Secondary access, Transcatheter aortic valve replacement

## Abstract

**Background:**

Transcatheter aortic valve replacement (TAVR) is a minimally invasive procedure with associated risks that are influenced by the choice of secondary vascular access used. The impact of transradial secondary access (TRSA) compared to transfemoral secondary access (TFSA) on adverse events remains uncertain. Therefore, we conducted an updated meta-analysis to compare procedural complications between TRSA and TFSA in TAVR.

**Methods:**

We systematically searched PubMed, Scopus, and the Cochrane Library for studies comparing TRSA and TFSA in patients undergoing TAVR. The primary endpoints were 30-day rates of each of the following: access-related bleeding, access-related vascular complications, stroke/transient ischemic attack (TIA), myocardial infarction (MI), acute kidney injury (AKI stage III or higher), and all-cause mortality. Leave-one-out sensitivity analyses and subgroup analyses stratified by primary access route were performed to assess the consistency of the findings.

**Results:**

Seven studies with 6327 patients were included, comprising of six observational studies and one randomized controlled trial TRSA was associated with significantly lower odds of mortality (OR 0.55, 95% CI [0.39, 0.78], p = 0.0007), stroke/TIA (OR 0.58, 95% CI [0.39, 0.87], p = 0.009), major/life-threatening bleeding (OR 0.50, 95% CI [0.30, 0.83], p = 0.008), and major vascular complications (OR 0.59, 95% CI [0.41, 0.85], p = 0.004). Additionally, we stratified outcomes for patients undergoing transfemoral primary access (TFPA) to determine whether the primary access route influences results. The transradial group demonstrated significantly lower odds of 30-day all-cause mortality (OR 0.49, 95% CI [0.28, 0.87], p = 0.01), and 30-day minor bleeding (OR 0.48, 95% CI [0.27, 0.86], p = 0.01).

**Conclusions:**

Key limitations included predominance of non-randomized studies and high heterogeneity in some outcomes. In patients undergoing TAVR, TRSA is associated with significantly lower complications compared to TFSA, suggesting it may be a superior alternative.

**Supplementary Information:**

The online version contains supplementary material available at 10.1186/s43044-025-00706-3.

## Introduction

Transcatheter aortic valve replacement (TAVR) is a minimally invasive technique that is gaining preference for managing severe aortic stenosis in patients at elevated risk for surgical valve replacement [1, 2]. The procedure involves the insertion of a prosthetic valve to substitute the damaged aortic valve. A catheter delivers the new valve, typically through the femoral artery. However, other access points may be used depending on the patient [3]. TAVR involves two vascular access points: primary access for valve delivery and a secondary access route for angiography, device placement, and hemodynamic monitoring [4, 5]. Despite being minimally invasive, vascular complications, bleeding, and strokes are frequent adverse consequences associated with TAVR. These risks are influenced by multiple factors, including age, comorbidities, and dual access sites [4, 5].

Historically, transfemoral secondary access (TFSA) has been the standard approach. However, emerging evidence suggests that transradial access secondary access (TRSA) is a better choice, particularly in reducing vascular and bleeding complications, as seen in percutaneous coronary interventions (PCI) and coronary angiography (CA) [6, 7]. In TFSA, the secondary catheter is inserted through the femoral artery, whereas TRSA uses the radial artery, offering a smaller caliber and more superficial access site [8]. Earlier studies have highlighted several benefits of secondary access involving the radial artery over the femoral artery, including its closer proximity to the coronary vessels and a reduction in access site-related incidence of complications [9].

Previous meta-analyses were limited by smaller sample sizes, heterogeneous outcome definitions, and the absence of recent large studies and the first randomized controlled trial (RCT) (*Versteeg* et al., 2024), [10] underscoring the need for an updated synthesis of evidence. Landmark studies such as *Junquera* et al. (2020) [11], *Grundmann* et al. (2024) [12], and Versteeg et al. (2024) [10] have provided important data linking TRSA to lower bleeding and vascular complications compared to TFSA, renewing interest in radial access during TAVR.

The impact of TRSA compared to TFSA on the adverse events in TAVR remains uncertain, with limited evidence in the literature comparing the two alternatives. To address the gaps in evidence, we conducted an updated systematic review and meta-analysis to robustly compare clinical outcomes between TRSA and TFSA, incorporating standardized endpoint definitions and stratified analyses by primary access route.

## Material and methods

### Study design

The study followed the Preferred Reporting Items for Systematic Reviews and Meta-Analyses (PRISMA) guidelines. The PRISMA checklist is provided in Supplementary Sect. 1. This review was preregistered with PROSPERO (ID: CRD42025643539).

### Search strategy

A systematic search of PubMed, Scopus, and Cochrane CENTRAL databases was performed to identify studies that compared TRSA and TFSA approaches in patients undergoing TAVR with any primary access modality. The search covered studies published from database inception through September 15, 2025. The full search string employed was as follows: “("Transcatheter Aortic Valve Replacement"[Mesh] OR TAVR OR "transcatheter aortic valve replacement" OR TAVI OR "transcatheter aortic valve implantation") AND (transradial OR radial OR "secondary access")”. Search results and specific database strategies are provided in Supplementary Sect. 2. In addition, a manual review of the references from all included studies, along with published systematic reviews and meta-analyses, was conducted to find any further relevant studies.

### Study selection

RCTs and observational studies that fulfilled the following criteria were included:

1. Participants who underwent TAVR using any primary access approach.
2. A comparison of TRSA and TFSA approaches for the specified endpoints.

### Exclusion criteria

1. Letters to editors, review papers, comments, posters, or abstracts.
2. Inaccessible English language full texts.

### Endpoints

The primary outcomes of interest were the 30-day rates of each of the following: all-cause mortality, major/life-threatening bleeding, minor bleeding, major vascular complications, minor vascular complications, myocardial infarction (MI), stroke/transient ischemic attack (TIA) and acute kidney injury (AKI) stage III or higher. For outcomes with missing or inconsistently reported data, efforts were undertaken to contact study authors for clarification. Studies that did not report specific 30-day outcomes, or for which data could not be obtained, were excluded from the corresponding outcome analyses.

### Screening and data extraction

The studies identified from the search were exported to Rayyan.ai, where duplicates were removed. Title and abstract screening, followed by full-text screening of the relevant studies, was conducted independently by two reviewers (E.F. and M.M.J.). In case of any conflicts, another reviewer (H.S.I.) was consulted. Two reviewers (E.F. and M.M.J.) independently extracted the data on a spreadsheet, and any conflicts were resolved by a third reviewer (H.S.I.). Information was gathered on study characteristics, such as study design and location, baseline characteristics of participants, including their age, gender, and comorbidities, and clinical outcomes. Studies were screened for overlapping patient cohorts to avoid duplicate data. Potential overlap was assessed by comparing study centers, years of patient enrollment, and geographic regions. Where multiple publications reported on the same cohort, only the most recent or most complete dataset was included.

### Quality assessment

Two reviewers (E.F. and R.H.S.) independently evaluated the risk of bias in the included studies. The Newcastle–Ottawa Scale (NOS) [13] was used for observational studies, whereas the Cochrane risk-of-bias tool for randomized trials (RoB-2) was utilized for the included RCT [14]. A third reviewer (H.S.I.) was consulted in case of any conflicts. Funnel plots and Egger’s test were not conducted, as these methods require a minimum of 10 studies per outcome to provide reliable results [14].

### Statistical analysis

Review Manager v5.4.1 (Cochrane Collaboration) was used to perform all statistical analyses [14]. Where available, adjusted or propensity-matched data were preferentially used. Otherwise, unadjusted data were included for synthesis.

Odds ratios (ORs) with 95% confidence intervals (CIs) were used to compare the effects of secondary access sites for categorical endpoints. Given the variability in methodologies across the included studies, ORs with 95% CIs were calculated using the random-effects Mantel–Haenszel model for dichotomous variables. A random-effects model was used as the populations across the included studies were assumed to differ. Forest plots were created to visualize the results.

Heterogeneity was assessed using Higgins I^2^ statistics. We used a cutoff of 50%, with a higher I^2^ value indicating greater heterogeneity and an I^2^ of less than 50% suggesting greater similarity among studies. All tests were two-sided, with a P-value of ≤ 0.05 being considered statistically significant. For outcomes with substantial heterogeneity (I^2^ ≥ 50%), a leave-one-out sensitivity analysis was conducted to evaluate the influence of individual studies on the pooled effect estimates. Subgroup analyses were performed when data were available, including analyses stratified by primary access site, bleeding definitions, and whether the patient populations were adjusted or unadjusted in the included studies.

## Results

### Study selection and characteristics

**Fig. 1:**
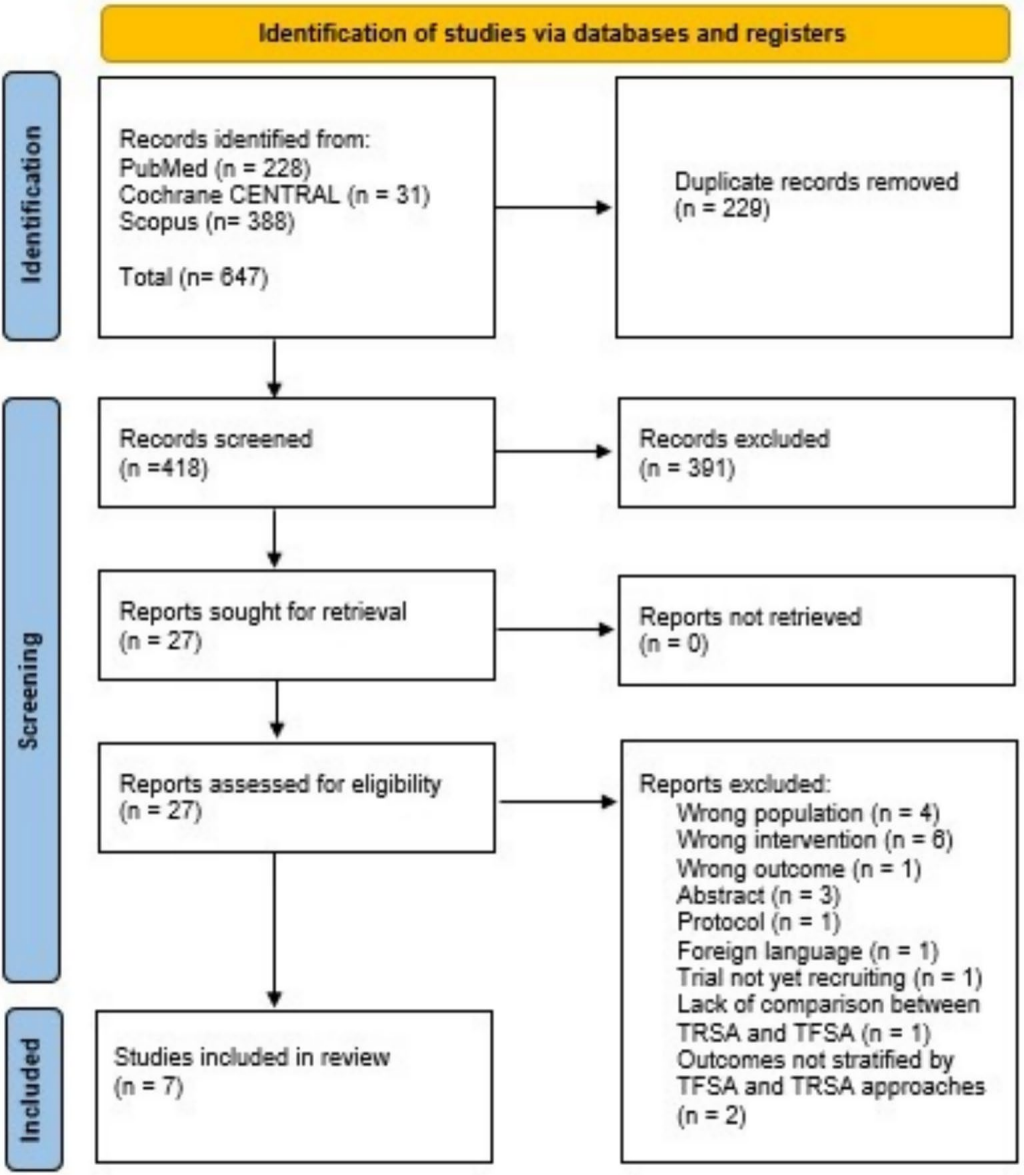
PRISMA flowchart summarizing the study identification and screening process for the systematic review and meta-analysis

Our search strategy identified 647 records. After duplicate removal and screening studies against the inclusion criteria, 27 remained for eligibility assessment. Of these, 6 observational cohorts [6, 11, 12, 15–17] and 1 RCT [10] were included **(**Fig. 1**)**, comprising a total of 6,327 patients who underwent TAVR: 4243 (67.1%) with TFSA and 2084 (32.9%) with TRSA.

Study characteristics are outlined in Supplementary Table 4.1. The recruitment period spanned an extensive timeframe from 2007 to 2023. Two of the studies were multicenter [10, 11], and all studies were carried out in Europe and North America. All studies reported the co-primary outcomes comparing TRSA and TFSA; however, only bleeding endpoints could specifically be stratified by secondary access type for the RCT, as other outcomes were potentially confounded by venous access.

The definitions of mortality, stroke/TIA, AKI, MI, vascular complications, and bleeding events were based on the Valve Academic Research Consortium-2 (VARC-2) criteria [18] in 5 studies, the original Valve Academic Research Consortium (VARC) criteria [19] in 1 study, and Valve Academic Research Consortium-3 (VARC-3) [20] and Bleeding Academic Research Consortium (BARC) [21] criterion in the RCT. Four studies [10, 12, 15, 16] reported solely on transfemoral primary access (TFPA), which involved the use of the femoral artery only. Three studies [6, 11, 17] reported on both TFPA, and non-transfemoral primary access (non-TFPA) approaches, with the latter encompassing subclavian, transapical, transaortic, transcarotid, and transcaval routes. However, *Junquera *et al*.* [11] had no data on the TFPA group separately. We conducted two separate analyses: one assessing outcomes across all primary access sites and another specifically evaluating TFPA. The four studies that reported data exclusively on TFPA were also included in the analysis of all primary access sites.

Supplementary Table 4.2 compares key patient characteristics. The mean age of the overall population was 80.9 years, with nearly half (47.9%) being females. Society of Thoracic Surgeons Predicted Risk of Mortality (STS-PROM) scores varied from 3.0% to 9.0%. The overall prevalence of hypertension and diabetes was 74.9% and 27.1%, respectively. Atrial fibrillation was present in 30.59% of the population on average, while the proportion of patients with prior coronary artery bypass graft (CABG) history ranged from 4.1% to 39.0%.

### Pooled analysis for all primary access

**Fig. 2:**
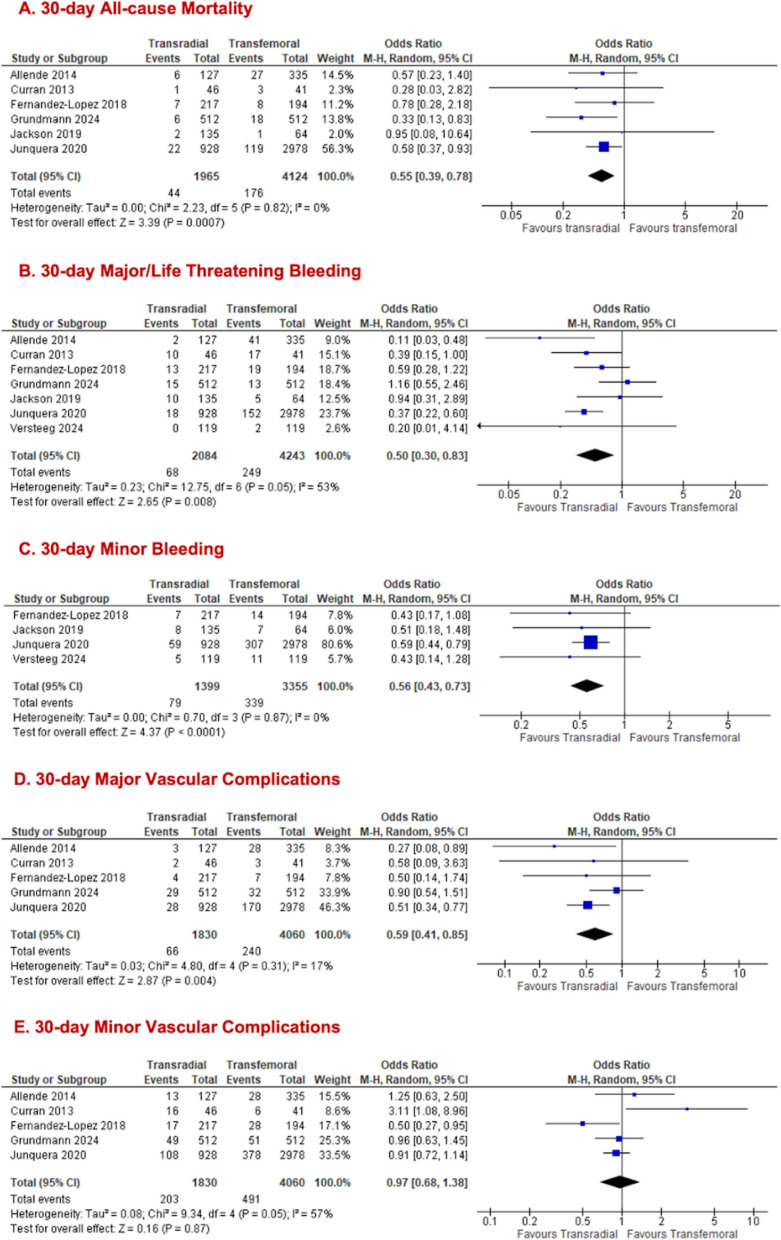
Forest plots comparing transradial and transfemoral access in all primary access TAVR for (**A**) all-cause mortality; (**B**) major/life-threatening bleeding; (**C**) minor bleeding; (**D**) major vascular complications; (**E**) minor vascular complications

Among patients undergoing TAVR procedures via all primary access sites, TRSA was associated with significantly lower 30-day all-cause mortality compared to TFSA (TRSA = 1965; TFSA = 4124; n = 6; 2.2% vs 4.3%; OR 0.55, 95% CI [0.39, 0.78], p = 0.0007, I^2^ = 0%; Fig. 2A). The incidence of the outcome of 30-day major/life-threatening bleeding was significantly lower in the TRSA arm (TRSA = 2084; TFSA = 4243; n = 7; 3.3% vs 5.9%, OR 0.50; 95% CI [0.30, 0.83]; p = 0.008; Fig. 2B) and heterogeneity was moderate (I^2^ = 53%). Removal of *Grundmann *et al*. (2024)* [13] in the sensitivity analysis for 30-day major/life-threatening bleeding improved the heterogeneity to 26% (Supplementary Fig. 5.1a), while preserving the significant results. Similarly, the odds of developing 30-day minor bleeding were significantly lower with TRSA (5.6%) compared to the TFSA (10.1%) arm (TRSA = 1399; TFSA = 3355; n = 4; OR 0.56; 95% CI [0.43, 0.73]; p < 0.0001; I^2^ = 0%; Fig. 2C). 30-day major vascular complications were significantly lower with TRSA than TFSA (TRSA = 1830; TFSA = 4060; n = 5; 3.6% vs 5.9%; OR 0.59; 95% CI [0.41, 0.85]; I^2^ = 17%; Fig. 2D). In contrast, no significant difference was observed between both arms for 30-day minor vascular complications (TRSA = 1830; TFSA = 4060; n = 5; 11.1% vs 12.1%; OR 0.97, 95% CI [0.68, 1.38], p = 0.87, I^2^ = 57%; Fig. 2E). Removal of Curran et al. (2013) [15] in the sensitivity analysis for 30-day minor vascular complications improved the heterogeneity to 27% while preserving the non-significant results. (Supplementary Fig. 5.1b).

**Fig. 3:**
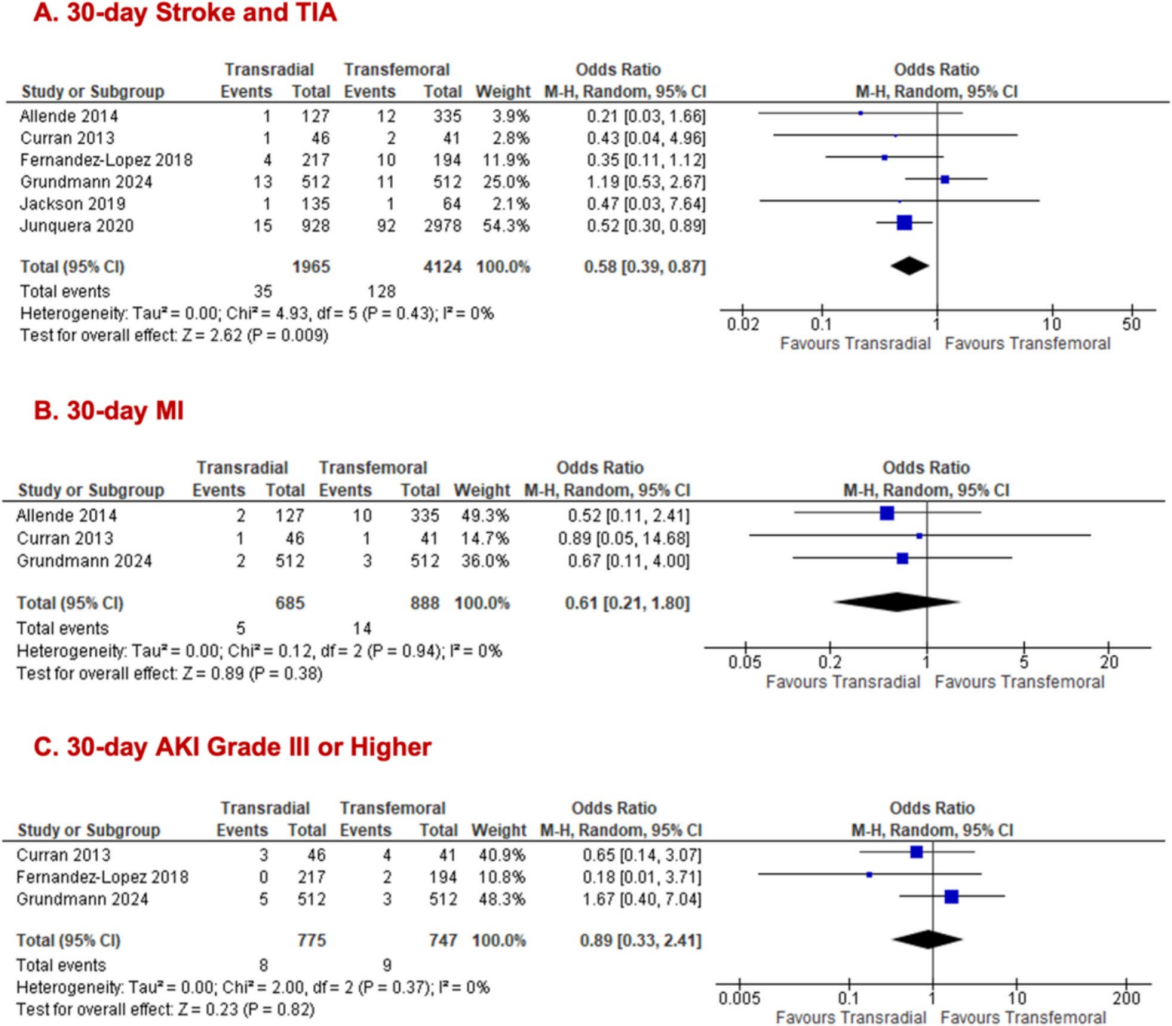
Forest plots comparing transradial and transfemoral access in all primary access TAVR for (**A**) stroke/TIA; (**B**) MI; (**C**) AKI grade III or higher

The odds of developing 30-day stroke/TIA were lower in the TRSA (1.8%) than in the TFSA (3.1%) arm (TRSA = 1965; TFSA = 4124; n = 6; OR 0.58; 95% CI [0.39, 0.87]; p = 0.009; I^2^ = 0%; Fig. 3A). The odds of developing 30-day MI (TRSA = 685; TFSA = 888; n = 3; 0.7% vs 1.6%; OR 0.61; 95% CI [0.21, 1.80]; p = 0.38; I^2^ = 0%; Fig. 3B) or 30-day AKI grade III or higher (TRSA = 775; TFSA = 747; n = 3; 1.0% vs 1.2%; OR 0.89; 95% CI [0.33, 2.41]; p = 0.82; I^2^ = 0%; Fig. 3C) were not significantly different between groups.

### Pooled analysis for TFPA

**Fig. 4:**
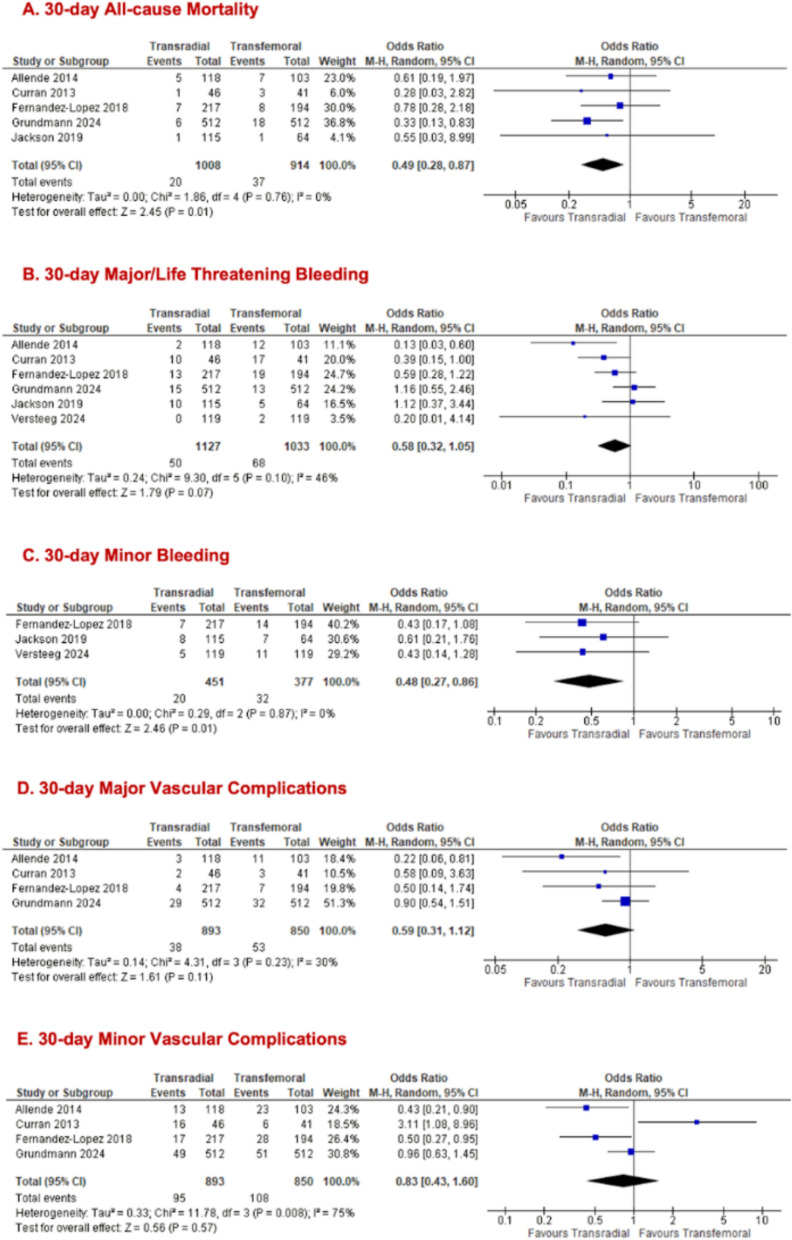
Forest plots comparing transradial and transfemoral access in only TFPA TAVR for (**A**) all-cause mortality; (**B**) major/life-threatening bleeding; (**C**) minor bleeding; (**D**) major vascular complications; (**E**) minor vascular complications

**Fig. 5:**
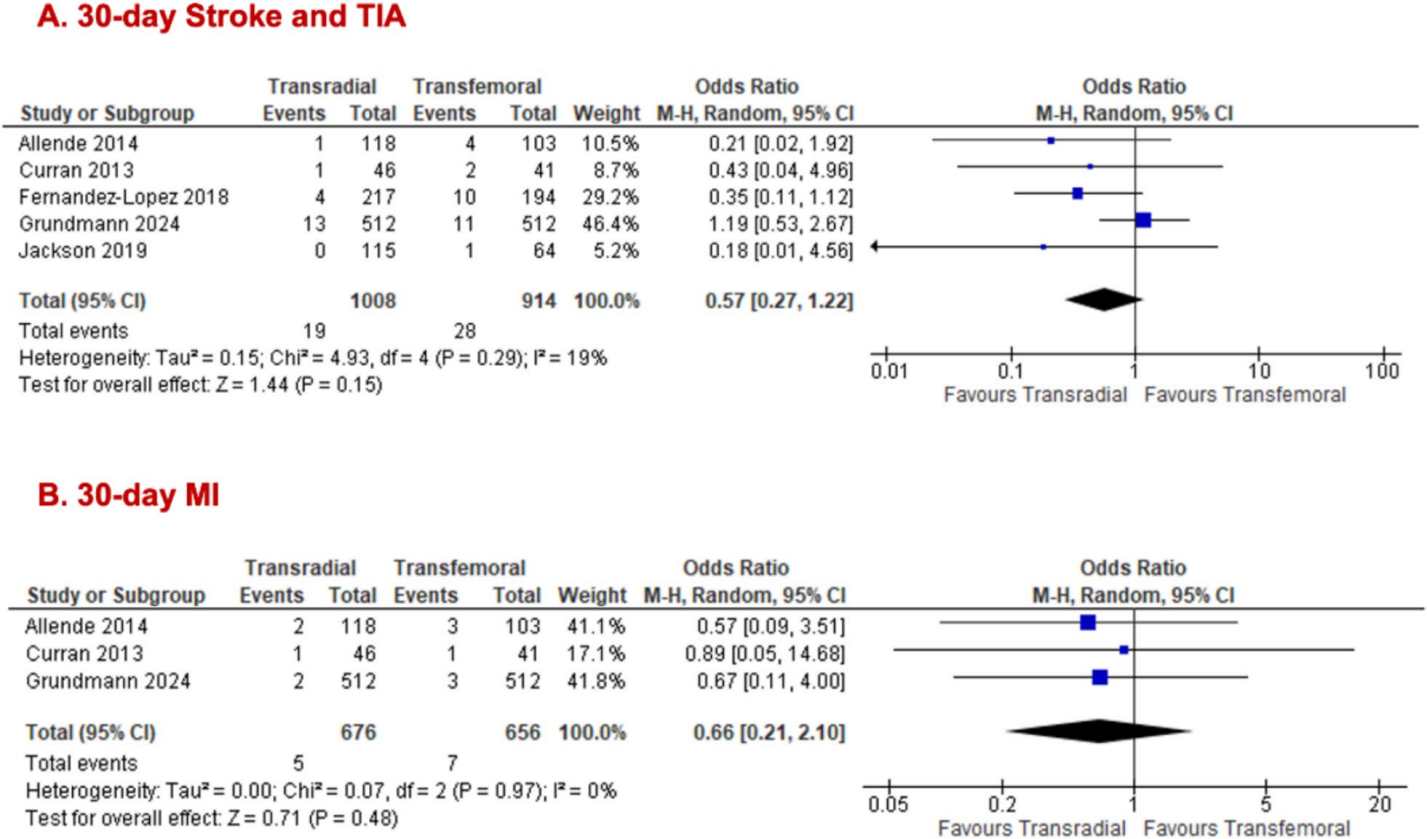
Forest plots comparing transradial and transfemoral access in only TFPA TAVR for (**A**) stroke/ TIA; (**B**) MI

We stratified outcomes of interest by populations who solely underwent TFPA to ascertain whether the primary access route influences results. The transradial group demonstrated significantly lower odds of 30-day all-cause mortality (TRSA = 1008; TFSA = 914; = 5; 2.0% vs 4.0%; OR 0.49, 95% CI [0.28, 0.87], p = 0.01, I^2^ = 0%; Fig. 4A). While no significant difference was noted for 30-day major/life-threatening bleeding between both arms (TRSA = 1127; TFSA = 1033; n = 6; 4.4% vs 6.6%; OR 0.58, 95% CI [0.32, 1.05], p = 0.07, I^2^ = 46%; Fig. 4B), there was a significant reduction in 30-day minor bleeding for TRSA (TRSA = 451; TFSA = 377; n = 3; 4.4% vs 8.5%; OR 0.48, 95% CI [0.27, 0.86], p = 0.01, I^2^ = 0%; Fig. 4C) was observed. All the following outcomes showed no significant differences between TRSA and TFSA groups: 30-day major vascular complications (TRSA = 893; TFSA = 850; n = 4; 4.3% vs 6.2%; OR 0.59, 95% CI [0.31, 1.12], p = 0.11, I^2^ = 30%; Fig. 4D), 30-day minor vascular complications (TRSA = 893; TFSA = 850; n = 4; 10.6% vs 12.7%; OR 0.83, 95% CI [0.43, 1.60], p = 0.57, I^2^ = 75%; Fig. 4E), 30-day stroke/TIA (TRSA = 1008; TFSA = 914; n = 5; 1.9% vs 3.1%; OR 0.57, 95% CI [0.27, 1.22], p = 0.15, I^2^ = 19%, Fig. 5A), and 30-day MI (TRSA = 676; TFSA = 656; n = 3; 0.7% vs 1.1%; OR 0.66, 95% CI [0.21, 2.10], p = 0.48, I^2^ = 0%; Fig. 5B). The forest plot for AKI grade III and higher in the TFPA cohort alone was identical to the one previously reported (Fig. 3C) in the all primary access cohort, as the same studies contributed data for this outcome. Removal of Curran et al. (2013) [15] in the sensitivity analysis for 30-day minor vascular complications improved the heterogeneity to 59% while preserving the non-significant result. (Supplementary Fig. 5.1c).

### Subgroup analyses

Subgroup analyses were performed according to standardized endpoint definitions (VARC-1, VARC-2, VARC-3, and BARC), whether studies carried out statistical adjustment, and by study design (observational studies vs. RCTs). When stratified by endpoint definitions, no statistically significant differences were observed between subgroups in relation to overall outcomes, except for 30-day minor vascular complications in the TFPA group (P = 0.003). In the adjustment-based analyses, most outcomes showed no significant differences between subgroups, except for 30-day minor vascular complications, which were significant in both the overall primary access group (P = 0.02) and the TFPA group (P = 0.008). When stratified by study design, no statistically significant differences were observed between subgroups across the evaluated outcomes. However, in the TFPA subgroup, the statistical significance for minor bleeding diminished in the individual subgroups as opposed to the overall significant results (observational = OR 0.50, 95% CI [0.25, 1.00], p = 0.05; RCT = OR 0.43, 95% CI [0.14, 1.28], p = 0.13). These findings should be interpreted with caution, as the unequal distribution of studies across subgroups may have influenced the results. All relevant forest plots have been provided in the Supplementary Sect. 5.2–4.

### Risk of bias assessment

The quality of the observational studies was evaluated using the NOS tool, with 5 studies [11, 12, 15–17] rated as high quality and 1 as moderate quality [6]. Participant selection, outcome assessments, and comparability were consistently well-managed across studies. The RoB-2 assessment for the included RCT indicated an overall low risk of bias, hence providing high confidence in the trial's validity and results. Plots generated using the risk-of-bias visualization tool [22] provide an overview of the quality assessments for all the studies, with further information available in Supplementary Sect. 3.

## Discussion

Our updated meta-analysis revealed that the odds of developing 30-day all-cause mortality, stroke/TIA, major/life-threatening bleeding, minor bleeding, and major vascular complications in patients undergoing TRSA are significantly lower than those undergoing TFSA, with the analysis conducted across all sites of primary access. However, the odds of developing MI, AKI grade ≥ III, and minor vascular complications were comparable between groups. Analysis of patients undergoing TFPA only revealed significantly lower odds of developing 30d all-cause mortality and minor bleeding, while the odds of developing other outcomes including stroke/TIA, MI, major or life-threatening bleeding, major and minor vascular complications, and AKI grade ≥ III, were similar in both groups.

The decreased benefit of TRSA in the TFPA subgroup, compared with the overall all primary access analysis, can be attributed to several factors. Randomized trials [23, 24] have shown that mortality is higher with nonfemoral primary access TAVR approaches (e.g., transapical, transaortic) than with surgical aortic valve replacement (SAVR), likely reflecting the higher comorbidity risk and more severe vascular disease in patients who are not candidates for transfemoral access. Consistent with this, the 2020 ACC/AHA [25] valvular heart disease guidelines recommend SAVR or palliative management for patients in whom vascular anatomy precludes TF TAVR (Class I, Level A). Hence in the all-access analysis, inclusion of higher-risk non-TF cases may have amplified the apparent impact of TRSA, making reductions in mortality, bleeding, and vascular complications appear more pronounced. This attenuation may reflect that TFPA patients are already selected for favorable vascular anatomy and lower comorbidity burden, leaving limited scope for TRSA to further reduce complications.

Moreover, the TFPA subgroup includes fewer patients and events, reducing statistical power and limiting the ability to detect differences that were significant in the larger pooled analysis. Residual confounding in observational studies—arising from differences in patient selection or unmeasured clinical factors—may disproportionately influence outcomes in non-transfemoral primary access cases. For example, in Allende et al. [6], 69.2% of non-transfemoral primary access patients were assigned to the TFSA group, potentially exaggerating the apparent benefit of TRSA in the all-access analysis.

Compared to previously published meta-analyses [26–28], our findings align with the general conclusion that TRSA is linked to significantly reduced odds of complications in comparison to TFSA. This meta-analysis includes the first-ever randomized trial by Versteeg et al. [10] comparing TFSA and TRSA in TAVR. Though limited only to access-related bleeding outcomes, it provides high-quality evidence on one of the most common [29] and prognostically [30] important complications after TAVR. We also included the propensity-score–matched study by Grundmann et al. [13], which is the largest cohort-based study on this subject after Junquera [11]. By incorporating these newer studies, our meta-analysis benefits from a larger pooled sample and strengthens the evidence base for assessing procedural complications in contemporary TAVR.

Our findings for all primary access sites align closely with those of the meta-analysis by Radhakrishnan et al. [26], with both analyses showing significantly lower odds of all-cause mortality, stroke/TIA, major or life-threatening bleeding, and major vascular complications with TRSA compared to TFSA. However, a notable divergence was observed in the TFPA subgroup. In our study, the odds of developing most outcomes in this subgroup did not significantly differ between the TRSA and TFSA arms However, TRSA was associated with significantly lower odds of all-cause mortality and minor bleeding. This contrasts sharply with the findings of Radhakrishnan et al. [26], who reported non-significant results for mortality odds but found that TRSA is associated with significantly lower odds of stroke/TIA and major or life-threatening bleeding compared to TFSA in this subgroup. This difference likely reflects our larger pooled sample size and use of more contemporary bleeding definitions (e.g. VARC-3 in newly included studies). Collectively, our analysis refines prior conclusions, suggesting that TRSA may lower mortality but does not consistently protect against stroke or major bleeding in TFPA patients.

Our analysis also addresses the ambiguity in data synthesis methods observed in previous meta-analyses. For example, Das et al. assessed outcomes exclusively in patients undergoing TFPA but combined the results of Junquera et al., which included patients from all primary access sites, with studies focusing solely on TFPA. Therefore, we conducted a comprehensive analysis, first evaluating the outcomes of all primary access sites, followed by a subgroup analysis focusing specifically on the outcomes of TFPA. In addition, two previous meta-analyses [27, 28] included a study by Lefevere et al. [31], which we, like Radhakrishnan et al. [26], excluded from our analysis. This study compared a standardized “FAST” protocol, characterized by local anesthesia, echo-guided femoral access, radial secondary access, and LV wire pacing, with a heterogeneous standard arm. Since some control patients underwent TRSA but were classified in the standard arm for not meeting all FAST criteria, including this study could introduce misclassification bias when specifically comparing TRSA and TFSA. Building on prior meta-analyses [27, 28], we refined AKI and MI estimates by limiting inclusion to studies with stratified AKI or non-zero event data, thereby ensuring consistent outcome definitions. Notably, whereas previous analyses reported a significant association for AKI [27] but not MI [28], our findings indicate no significant differences for either outcome.

However, this meta-analysis pooled data from both randomized and observational studies. While inclusion of observational evidence enhances overall sample size and statistical power, it introduces potential confounding, as non-randomized studies are intrinsically susceptible to selection bias and unmeasured covariates. Consequently, pooled effect estimates should be interpreted with caution.

An important consideration in TAVR studies is the rate of secondary access failure or crossover, which could potentially influence study results. Specifically, 5 of 132 planned TRSA patients (3.8%) in Allende [6] with failed radial access were excluded, 7 of 122 planned transradial cases in Jackson [17] were converted to femoral but analyzed in the femoral cohort, 19 of 952 transradial attempts in Grundmann [12] required conversion to femoral access, and in Versteeg []10, 9 arterial access failures occurred, but patients were retained in the intention-to-treat analysis. Curran and FL [15, 16] did not report crossover or failure rates. Overall, rates of secondary access failure or crossover were generally low across studies, suggesting limited impact on procedural outcomes and complication rates.

A meta-analysis involving 30,096 patients undergoing PCI and CA demonstrated that RA significantly reduces the risks of mortality, bleeding, and vascular complications relative to femoral access [32]. Consistent with this, a recent study in patients with chronic coronary disease undergoing PCI reported that FA was associated with more complex procedures, lower procedural success, and higher rates of major bleeding compared to RA[33]. Similarly, utilizing secondary radial access in TAVR presents an opportunity to enhance patient outcomes by mitigating all-cause mortality, bleeding, and vascular complications.

The RA approach offers several benefits compared to the femoral access approach. It provides better control over bleeding due to the radial site being easier to compress [34], and it avoids the need to access both femoral arteries, which can reduce the risk of vascular complications [15]. However, RA comes with limitations, such as challenges in navigating anatomical variations or dealing with tortuous and calcified radial arteries, particularly in elderly patients [35]. Additionally, the use of larger catheter diameters may raise the likelihood of radial artery occlusion, and it does not accommodate certain interventional equipment, including intra-aortic balloon pumps or larger 8F catheters required for complex procedures [34, 36].

On the other hand, the femoral access approach is preferred in situations where RA is complicated by anatomical factors or when managing iliofemoral vascular complications [37]. It involves the use of balloons, stents, and longer guidewires, which may not be present in all centers [38]. However, the transfemoral route is associated with an increased likelihood of bleeding and vascular complications. Improvements in equipment design have enhanced the safety of the radial approach, but in cases of insufficient radial access or high-risk bleeding scenarios, the femoral artery remains a more suitable option to manage complications effectively.

Vascular and bleeding complications significantly impact TAVR outcomes, being linked to higher mortality, poorer quality of life, extended hospital stays, and higher expenses [39–44]. The smaller vessel diameter associated with radial artery access may contribute to reduced complications and bleeding at the vascular insertion site [45].

Patient selection is a key determinant of secondary access choice in TAVR. TRSA may be most feasible in patients with favorable radial anatomy and low risk of arterial complications, whereas femoral access remains preferred in patients with tortuous or calcified radial arteries, inadequate vessel diameter, or need for larger interventional equipment. In the context of TFPA patients, who are generally selected for favorable vascular anatomy and lower comorbidity burden, the additional benefit of TRSA appears limited, as procedural risks are already low. Conversely, patients with higher-risk profiles or challenging femoral anatomy may derive greater benefit from TRSA, but the apparent advantage observed in all-access analyses could be exaggerated by uneven baseline risk distribution, and further randomized studies are needed to identify which patient populations gain the most clinical benefit.

Periprocedural hemostatic control during large-bore vascular access may influence access-site bleeding and vascular complication rates in TAVR. Evidence from the multicenter ACE-PROTAVI trial [46] in patients undergoing transfemoral TAVR demonstrated that routine protamine administration significantly improved hemostasis while reducing time to hemostasis and minor vascular complications. Complementing this, a meta-analysis of six RCTs [47] in patients undergoing percutaneous cardiac interventions found increased hemostasis success rate, without an increase in major bleeding, stroke, or mortality, potentially supporting protamine as a safe strategy to enhance hemostatic control in TAVR. Moreover, a recent meta-analysis of 3,117 patients [48] comparing continued versus interrupted oral anticoagulation found no significant differences in major bleeding outcomes or major vascular complications, though minor bleeding was slightly higher with continued therapy. Similarly, ultrasound-guided arterial cannulation has been shown to improve first-attempt success and reduce complications, highlighting the potential benefits of imaging-guided access in procedures such as TAVR [49].

Stroke/TIA is another significant complication following TAVR, that has been correlated with a heightened risk of all-cause mortality in comparison to patients who do not experience stroke [50]. Our meta-analysis demonstrates that TRSA, regardless of the primary access site, reduces the odds of stroke compared to TFSA. The exact reasons why TRSA may reduce cerebrovascular events remain unclear and previous studies have described conflicting results regarding its association with access sites [51]. Elderly patients undergoing TAVR are likely to have atherosclerosis due to the age-related progression of vascular disease [52]. A previous study demonstrated that direct manipulation of the aortic arch during endarterectomy in elderly patients with atheromas was associated with an increased intraoperative stroke incidence [53]. Unlike TFSA, which requires catheter navigation through the descending aorta, TRSA minimizes arch manipulation potentially reducing the risk of embolization and cerebrovascular events.

AKI is another significant complication that adversely impacts mortality in TAVR patients, as demonstrated by several studies [54–57]. A nationwide Danish study reported that approximately 5% of TAVR patients develop AKI, with an increased one-year rate of dialysis during hospitalization [58]. However, our findings indicate that TRSA does not reduce the odds of AKI grades III and higher compared to TFSA.

Future randomized clinical trials, VARC-TAVI (NCT06177392) [58], SAFER-TAVI (NCT06284837) [33, 59] and CN-02829855 [56], comparing transfemoral and transradial access are expected to assess additional outcomes beyond bleeding, therefore providing further insight into the comparative effects of TAVR based on secondary access sites. More broadly, randomized trials are needed to validate the findings of this meta-analysis and strengthen the evidence base for selecting optimal secondary access strategies.

Our study presents certain limitations that must be acknowledged. Six of the seven studies included were non-randomized, increasing selection bias and limiting causal inference, and the single RCT contributed a small sample size and included only reported bleeding outcomes. High heterogeneity was observed in some outcomes, particularly 30-day major bleeding and minor vascular complications, which may reflect differences in patient populations, study design, or outcome reporting. Furthermore, differences in operator experience, learning curves associated with transradial access and center volume, may have influenced outcomes. Access-related bleeding and vascular complications were inconsistently reported, and bleeding criteria varied, with older studies using VARC-2 and newer ones VARC-3 criteria, limiting standardization. Most studies were conducted in Europe or North America, limiting generalizability to other populations with different comorbidity profiles or vascular anatomy. Confounding by indication also remains possible, as patients undergoing non-transfemoral access may represent inherently higher-risk cohorts. Despite adding two studies, the TFPA subgroup remained small. Notably, unlike the previous meta-analysis [26], our findings showed a marked difference in the results between TFPA and all-access-site subgroups, highlighting the need for further research to clarify TRSA’s benefits over TFSA in TFPA. Long-term outcomes were not assessed, precluding evaluation of the sustained effects of TRSA and TFSA access strategies.

## Conclusion

Our meta-analysis suggests that TRSA is associated with lower 30-day mortality, stroke/TIA, bleeding, and vascular complications compared with TFSA across most access sites, while in the TFPA subgroup, the benefit appeared limited to mortality and minor bleeding. These associations, although strengthened by inclusion of recent studies, remain subject to potential residual confounding and heterogeneity in outcome definitions. Future randomized trials will be essential to confirm access-site safety and efficacy. Future research should refine patient selection, optimize techniques, and assess long-term outcomes to improve TAVR access-site decisions.

## Supplementary Information

Supplementary file 1.

## Data Availability

No datasets were generated or analysed during the current study.

## Abbreviations

AKI: Acute kidney injury
BARC: Bleeding academic research consortium
BMI: Body mass index
CABG: Coronary artery bypass graft
CA: Coronary angiography
CAD: Coronary artery disease
CI: Confidence interval
CVA: Cerebrovascular accident
HTN: Hypertension
I^2^: Higgins’ I-squared (measure of heterogeneity)
MI: Myocardial infarction
NOS: Newcastle–Ottawa scale
NR: Not Reported
NYHA: New York heart association
OR: Odds ratio
PCI: Percutaneous coronary intervention
PRISMA: Preferred reporting items for systematic reviews and meta-analyses
PROSPERO: International prospective register of systematic reviews
PSM: Propensity score matching
RA: Radial artery
RCT: Randomized controlled trial
RoB-2: Risk of bias tool 2
SAVR: Surgical aortic valve replacement
SD: Standard deviation
STS-PROM: Society of thoracic surgeons predicted risk of mortality
TAVR: Transcatheter aortic valve replacement
TFPA: Transfemoral primary access
TFSA: Transfemoral secondary access
TIA: Transient ischemic attack
TRSA: Transradial secondary access
VARC: Valve academic research consortium
